# Mass spectrometry-based metabolomics of volatiles as a new tool for understanding aroma and flavour chemistry in processed food products

**DOI:** 10.1007/s11306-019-1493-6

**Published:** 2019-03-12

**Authors:** Carmen Diez-Simon, Roland Mumm, Robert D. Hall

**Affiliations:** 1 Laboratory of Plant Physiology, Wageningen University and Research, Droevendaalsesteeg 1, Wageningen, The Netherlands; 2 Wageningen Research, Wageningen University and Research, Droevendaalsesteeg 1, Wageningen, The Netherlands; 3 Netherlands Metabolomics Centre, Einsteinweg 55, Leiden, The Netherlands

**Keywords:** Mass spectrometry, Gas chromatography, Maillard reaction, Food processing, Flavour chemistry, Volatiles, Process flavours

## Abstract

**Background:**

When foods are processed or cooked, many chemical reactions occur involving a wide range of metabolites including sugars, amino acids and lipids. These chemical processes often lead to the formation of volatile aroma compounds that can make food tastier or may introduce off-flavours. Metabolomics tools are only now being used to study the formation of these flavour compounds in order to understand better the beneficial and less beneficial aspects of food processing.

**Aim of review:**

To provide a critical overview of the diverse MS-based studies carried out in recent years in food metabolomics and to review some biochemical properties and flavour characteristics of the different groups of aroma-related metabolites. A description of volatiles from processed foods, and their relevant chemical and sensorial characteristics is provided. In addition, this review also summarizes the formation of the flavour compounds from their precursors, and the interconnections between Maillard reactions and the amino acid, lipid, and carbohydrate degradation pathways.

**Key scientific concepts of review:**

This review provides new insights into processed ingredients and describes how metabolomics will help to enable us to produce, preserve, design and distribute higher-quality foods for health promotion and better flavour.

## Introduction

Flavours are perceived by the aroma and taste receptors in the nose and the mouth, respectively. There are five base taste types (sweet, salty, sour, bitter and umami), which are supplemented by the specific perception of many different aroma compounds. The most important characteristic of flavour is the aroma, and the contribution of the odorous volatile substances. Flavour chemistry is the study of the chemical compounds considered to cause an aroma and/or taste. By performing taste panel studies, we are able to discover whether a food product tastes or smells good, bad or ‘better than others’. However, it is still not well understood which (types of) compound(s) are imparting these characteristics (Sucan and Weerasinghe 2005) and how the interaction between different components including chemical composition, formulation, temperature, etc. influence the overall consumer perception. The chemical groups that influence the aroma characteristics of food are mainly volatile. However, non-volatile compounds can also play important roles in the sensory aroma profile, either as flavour precursors or directly, as flavourings. These are related to the sweetness, bitterness, sourness, saltiness and umami sensations and so, they make a strong contribution to the flavour of processed foods. Amino acid components, nucleotides, phenolic compounds, organic sugars and fatty acids are all examples of chemical groups that can have a role in determining the overall flavour of processed food. However, non-volatile compounds are beyond the scope of this review. Here, we focus on the volatile/aroma chemistry of process flavours through the use of metabolomic tools.

In this review, we give particular emphasis to the application of, and potential for, metabolomics approaches to advance our knowledge specifically on process flavours and flavourings as widely used in the food industry. We shall also focus on ‘natural’ ingredients as nowadays food companies are giving increasing importance to new food experiences which increasingly specifically involve only ‘natural’ flavours, colours, and preservatives (Attokaran 2017). According to the European Union, an ingredient is considered natural when it has been entirely derived from a source material that is vegetable, animal, or microbiological in nature, and at the same time, has been created through traditional food preparation processes (EU Regulation 1334/2008 Article 16 clause 2, 16 December 2008).

### Food processing

Our modern lifestyle and the ever-growing global population have caused increased demands on the food processing industry. Food processing can be defined as the physical and/or chemical manipulation of raw food to generate products that can easily be prepared and served by the consumer. At the same time, processed foods can generally be more easily stored for longer periods, facilitating a broader availability to a global population. Preparation of food involves a great variety of processes; from mincing, to pasteurisation, to cooking, to fermentation, to packaging, etc. Thus, foodstuffs such as cheese, bread, breakfast cereals, tinned vegetables, savoury snacks, biscuits and milk are all processed foods.

Food quality can be influenced by the preparation process. This directly affects nutritional value and potential health benefits of food, as well as the sensory attributes (Tamanna and Mahmood 2015). Therefore, food processing can have both beneficial and detrimental effects. Beneficial effects include the improvement of digestibility and bioavailability of nutrients, inactivation of food-borne pathogens, toxins or other detrimental constituents, prolongation of shelf-life and the improvement of the texture, taste and smell (van Boekel et al. 2010). All these changes increase consumer attractiveness. On the other hand, processing can also induce deleterious effects, such as loss of vitamins and other nutrients, the formation of toxic compounds or of compounds conferring negative effects on flavour perception, texture or colour. A well-known example for instance, is acrylamide which has been classified as a probable carcinogen in humans (Tareke et al. 2000). During preparation at high temperature, acrylamides can be formed in many types of foods via Maillard reactions (Mottram et al. 2002) including fried potato products (Vinci et al. 2012). By identifying which chemical species directly contribute to flavour perception, food manufacturers gain a better mechanistic understanding of how to produce more palatable food through directing the formation of desirable flavour attributes and reducing the occurrence of undesirable ones.

Next to the basic raw materials used, other components are often added during processing for a wide range of reasons related to stability, appearance, flavour enhancement, aroma, etc. These additives can be constituted by single molecules such as so-called top notes as well as monosodium glutamate (MSG), sugar and organic acids or mixtures like protein hydrolysates or extracts. A specific group of ingredients are the so-called ‘reaction flavours’ or ‘process flavours’. These are complex mixtures which are often added to savoury products such as soups and sauces, but also to coffee, to modify taste and enhance specific sensory attributes in the final product (Sucan and Weerasinghe 2005). Process flavours have complex origins, related to spices, fruit (juice), vegetable (juice), yeast, herbs, bark, buds, dried roots, leaves or any other edible portions of a plant, or fermentation products. Processed foods therefore tend to have more complex biochemical profiles as compared to the fresh materials.

### Process flavours

For many decades, the food industry has tried to make plant-based food taste ‘meaty’ in support of the growing vegetarian and vegan communities. Cooked meat flavour has been one of the main focus points in processed food flavours (Kerth and Miller 2015). The most important step in creating meat flavour is the Maillard reaction. Maillard reactions are a complex group of chemical reactions that occur between amino acids and sugars. They trigger a great number of reactions that lead to the formation of flavour compounds, also characteristic of brown colour formation. The first commercial use of Maillard reactions to produce process flavourings took place in the 1960s at the Unilever subsidiary company Food Industries Ltd. A number of landmark patents were filed (May and Akroyd 1960; Morton et al. 1960), which protected the reactions of the key precursors of meat flavour. Prior to this, a savoury character was usually generated through the use of hydrolysed vegetable proteins (HVPs), spice blends or actual meat extracts. However, these materials did not infer the desired meaty flavour (Parker 2015) and were not appropriate for a vegan diet. Nowadays, alternative strategies to produce meat-type flavours are being developed by using natural yeast-based ingredients.

**Fig. 1:**
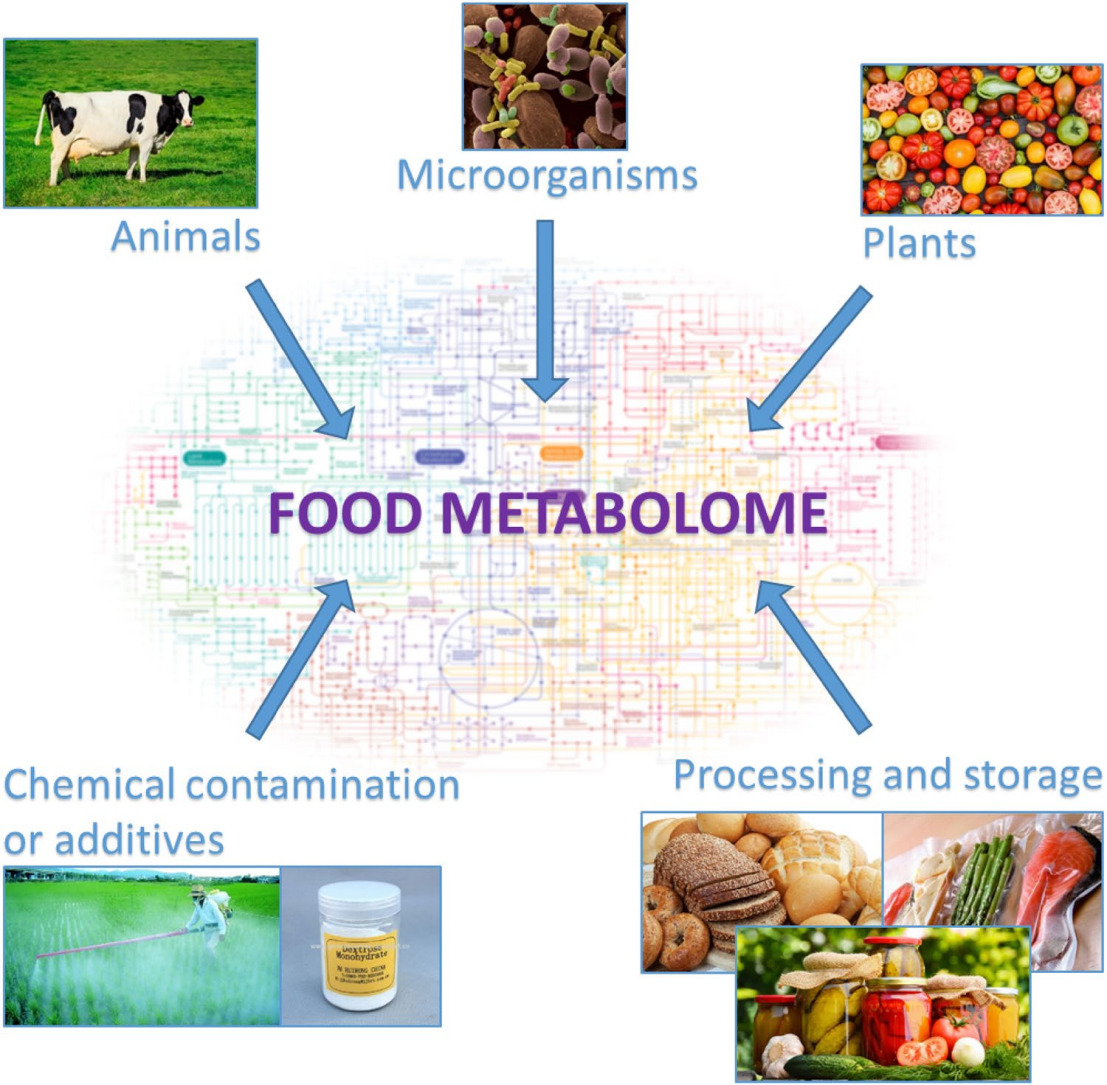
In terms of metabolites, food is a very complex material. Plant, animal and microbial materials cannot only be directly consumed but also often after highly-influential processing steps. Here the main sources of food metabolites are given which together constitute the food metabolome (modified from Johanningsmeier et al. 2016)

Yeast (*Saccharomyces cerevisiae*) has become a regular ingredient in the food industry. Some of the most outstanding ingredients now used for natural flavouring in process flavours are yeast extract-based products and yeast autolysates (In et al. 2005), particularly when a meaty aroma is required (Lin et al. 2014). These enhance the food flavour by imparting cheesy, meaty or savoury notes, but can also be used as texturizers, stabilizers and thickeners (Sucan and Weerasinghe 2005). Yeast extracts are also known for their nutritional benefits as they have a relatively high content of protein, vitamins (B1, B2 and nicotinic acid) and minerals (Sucan and Weerasinghe 2005). In addition, they can be used for salt reduction in processed food without compromising the ‘saltiness sensation’ of the food product (Batenburg and van der Velden 2011). By varying the autolytic conditions used in yeast processing, such as temperature and time, different meaty and savoury notes can be obtained to suit different industrial purposes (Ames 1994; Münch et al. 1997; Mahadevan and Farmer 2006). Components can be either already present in the starting materials or they are formed as a result of the processing strategy used (Fig. 1). Cooked foods develop characteristic flavours and colours, which are formed through complex series of reactions mainly related to Maillard reactions, lipid oxidation, and thermal degradation (Parker 2015). These reactions and their importance to flavour development are explained in detail throughout this review.

## Metabolomics in food processing

Metabolomics is defined as the comprehensive characterization of all the small molecules present in a biological sample and is used to compare accurately the metabolite profiles between groups of samples (Zhang et al. 2012). Small-molecule metabolites play a central role in food quality as they are often the coloured, fragrant or bioactive compounds contributing directly to nutritional value and to both positive sensory attributes as well as negative ones such as the so-called, off-flavours. The general application of metabolomics in food science and nutrition has been reviewed (Wishart 2008; Cevallos–Cevallos et al. 2009; Scalbert et al. 2009) and the importance of improvements in food analytical chemistry, such as high-resolution mass spectrometry and advanced statistical techniques to process the large data sets, have already been emphasised (Rubert et al. 2015). However, many limitations are still evident especially relating to the high complexity of the processed food matrices and the importance of low abundancy compounds with low aroma thresholds.

More than 20,000 compounds are known from food (http://www.Foodb.ca). To understand the relationship between food quality and processing, a complete analysis of the metabolites present in a food sample is needed (Thissen et al. 2011). State-of-the-art metabolomics comprises analytical platforms such as gas chromatography and liquid chromatography–mass spectrometry-based techniques (GC–MS and LC–MS) and nuclear magnetic resonance (NMR) spectroscopy. Each approach has its own advantages and disadvantages (which are beyond the scope of this review: see Johanningsmeier et al. 2016). Each can routinely be used to obtain metabolomic data sets due to their versatility, dynamic range, sensitivity, unique accessibility, etc. (Marshall and Powers 2017). Metabolomics has improved our capacity to analyse the overall metabolome as well as helping to perform pathway analysis and metabolite identification (Zhang et al. 2012; Van Duynhoven and Jacobs 2016; Marshall and Powers 2017). Here, we focus primarily on mass spectrometry (MS)—based techniques as these are the most widely used in food science.

Significant compositional changes occur during pre-harvest, post-harvest, and processing of foods. Metabolomics approaches have been widely exploited at each of these stages to help advance our knowledge of firstly, which components are present in which parts of our food materials and how they change or appear in time (Kim et al. 2016) and secondly, how specific processing strategies influence final composition (Tamanna and Mahmood 2015). Van Boekel et al. (2010) and Tamanna and Mahmood (2015) have discussed general aspects of food processing on nutritional components. However, we still need more knowledge of the other quality aspects not directly linked to nutrition.

Studying how processing affects sensorial properties of food ingredients used as flavourings is of specific importance. Plants such as onion (*Allium cepa*) are used for enhancing the flavour of many processed flavours due to the high content and variety of sulphur containing compounds. Processing operations such as drying/dehydration, high hydrostatic pressure, ohmic heating, etc. can influence both the abundance and composition of metabolites in the final product. Colina-Coca et al. (2013) reported a new approach using dynamic headspace (DHS) GC–MS for the analysis of volatile compounds in onion to evaluate how processing operations affect the final aroma profile. Metabolomics has also been used for the analysis of polysulphides, which are the primary compounds determining garlic flavour, derived from the degradation of allicin when garlic is cooked/processed (Tocmo et al. 2017). To evaluate the effects of processing on polysulphides, GC–MS and LC–MS analyses of garlic extracts revealed that shorter boiling times enrich linear polysulphides, especially trisulphides and allyl disulphides (next to garlic aroma, this confers meaty nuances to the taste of food already at 2 ppm; Burdock 2009).

There is currently limited metabolomics literature on flavours resulting from processing of yeast derived products. Yeast extracts were studied for the first time by Izzo and Ho (1991) and Ames and Elmore (1992), and later in more detail (Ames 1994; Münch et al. 1997). Sucan and Weerasinghe (2005) provide an overview of process and reaction flavours. Recently, Zhang et al. (2017) reported the analysis of ‘yeasty’ off-flavour volatiles from yeast extracts used as food ingredients. Volatiles have been the most studied flavour compounds although metabolomics has yet been poorly exploited for yeast product analyses.

### Two approaches: untargeted and targeted metabolomics

Metabolomics analyses have already proved valuable to the food industry for the analysis of the aroma of fresh [e.g. tomato (Thissen et al. 2011), melon (Allwood et al. 2014)] and processed [wine (Cozzolino 2016), or vegetable purees (Lopez-Sanchez et al. 2015)] materials. Metabolomics analysis can follow targeted or untargeted approaches (Patti et al. 2012). In untargeted analysis, the aim is to detect as many components from the matrix as possible in an unbiased manner. An untargeted approach is chosen to evaluate the overall metabolite profile of the studied system, without anticipating which (classes of) compounds are responsible for differences in the metabolic profiles. Technically, this is achieved by metabolic fingerprinting or profiling approaches (Hall 2006). A typical objective in metabolomics studies is the detection of biomarkers. Biomarkers in the food context are compounds that indicate a certain state or the perturbation of a metabolic system either by their presence or abundance change. Often, a combination of untargeted analysis followed by one or several targeted analyses in order to capture all the information is needed (Esslinger et al. 2014).

Targeted analyses rely on a priori knowledge of the class of metabolites that are expected to contribute to the (sensory) properties of interest (Scalbert et al. 2009).

However, food matrices are highly complex involving compounds with very different physical and chemical properties. Appropriate sample preparation methods and accounting for the influence of matrix effects are essential during data analysis and interpretation. Extraction of components has been traditionally done by using universal solvents (Patti et al. 2012). Solventless extraction of volatiles such as headspace technique is a fast, sensitive and economical alternative (Kataoka et al. 2000). Metabolites can also be analysed without extraction by using e.g. NMR (Van Duynhoven and Jacobs 2016), direct infusion MS (Baker et al. 2012), PTR-MS (Biasioli et al. 2011), infrared (IR) spectrometry (Aernouts et al. 2011), RAMAN Spectroscopy (Goodacre et al. 2018) and MS imaging (Matros and Mock 2013; Kadam et al. 2016) as well as the emerging SWATH-MS technology (Stolle et al. 2018). There is no singular method that allows for accurate, sensitive and complete reporting of all chemical species in a food sample. However, with new analytical developments, comprehensiveness in coverage continues to increase (Lopez-Sanchez et al. 2015). LC–MS-based methodologies have been proposed to be best suited for the identification of novel bioactive compounds in plant foods because of the compatibility of LC separation with the diversity of metabolites present (Johanningsmeier et al. 2016). GC–MS-based methods have been broadly applied for the analysis of food volatiles and may also be applied to the study of derivatised, non-volatile polar components such as mono- and di-saccharides, sugar alcohols, organic acids, amino acids, and long-chain fatty acids. Headspace techniques are now regularly being used to study the volatile aroma composition of food products. Each technique has its own advantages and limitations and these are highlighted in more detail for specific flavour compound groups in Sect. 4 (See also Table 1). For a more detailed overview of the wide range of methodologies we can refer to the many recent chapters in Antonio (2018).

**Table 1:** Examples of aroma/flavour related compounds analysed with different extraction techniques in combination with GC–MS during food processing

Volatile chemical group	Main sensory attributes	Formation pathways	Extraction technique	References
Aldehydes, alcohols and ketones	Fatty Herbal Fruity Nutty Earthy	- Fatty acid oxidation - Thermal degradation of carbohydrates - Thermal degradation of amino acids - Oxidation of carotenoids	SPME, SAFE LLE SBE Dimethylacetal derivatives	Xu et al. (2007), Gao et al. (2010), Chen et al. (2015) Zheng et al. (2013) Fan et al. (2011) Berdyshev (2011)
Organic acids and derived esters	Fruity sweet-like	Lipid metabolism	SDE	Lin et al. (2014)
N- and O- containing heterocyclic compounds				
Furan-derivatives	Caramel-like Fatty Nutty	- Thermal degradation of carbohydrates - Thermal degradation of amino acids - Maillard reaction - Oxidation of polyunsaturated fatty acids - Oxidation of carotenoids - Oxidation of Vitamin C	SPME AEDA	Feng et al. (2015), Seok et al. (2015) Kaneko et al. (2013), Poehlmann and Schieberle (2013)
Pyrazines	Roasted Nutty Cocoa Sweet	- Thermal degradation of amino acids - Maillard reaction	DHS, SDE	Lin et al. 2014
			SPME	Adams et al. 2008
S-containing compounds				
Alkyl sulphides and polysulphides	Cooked meat aroma Alliaceous Off-flavor	- Maillard reaction - Thermal degradtion of Vitamin B1	DHS SD, SDE, SPTE, SPME LLE	Colina-Coca et al. 2013 Lee et al. (2003), Murray (2001) Tocmo et al. (2017)
Thiophenes	Meaty aroma	- Maillard reaction - Thermal degradation of Vitamin B1 - Thermal degradation of nucleotides	DHS, SDE	Lin et al. (2014), Mahadevan and Farmer (2006)
Thiazoles and thiazolines	Roasted	Interactions between Maillard reaction products and lipid-derived aldehydes	DHS	Elmore et al. (1997), Shahidi et al. (2014)

*DHS* dynamic headspace, *LLE* liquid–liquid extraction, *SAFE* solvent-assisted flavour evaporation, *SBSE* Stir-bar sorptive extraction, *SD* steam distillation, *SDE* simultaneous distillation and extraction, *SPME* Solid-phase microextraction, *SPTE* solid-phase trapping solvent extraction

### The complexity of food matrices

The analysis of flavour compounds is challenging due to several factors including their high dynamic range, relevant presence at low concentrations (ppm, ppt), high range of polarity, extreme high volatility (high vapour pressures) and the instability of some flavour compounds in dynamic equilibria with other constituents of the food matrix (Dresow and Böhm 2009). Moreover, the complexity of the food matrix provides the greatest challenge. The physical and chemical interactions of all compounds present in a food matrix determine the ‘overall sensory experience’. This complexity sometimes incurs limitations to the extraction/separation methods (Scalbert et al. 2009) but since perceived flavour is not determined by a single component, the identification of the overall sensory-relevant metabolite profile, through untargeted analysis is needed to allow us to recognise which metabolite(s) or metabolic pathway(s) correlate with the food processing strategy and final product quality.

Lipids, proteins, and carbohydrates in the food matrix can positively or negatively affect the flavour of food products. The release of aroma compounds from foods is determined by the partition coefficient between the air phase and food matrix (and here also between the hydrophobic and hydrophilic phases). The nature of these interactions depends on the physicochemical properties of flavour compounds and food components (Jeleń et al. 2012). For instance, fat composition, concentration, emulsion characteristics and temperature, can each significantly modify interactions between lipids and small molecules (Piraprez et al. 1998; Guichard 2002). The retention of different aroma compounds in lipid matrices is strongly influenced by their molecular weight and chemical structure (Piraprez et al. 1998). Lipid oxidation produces a variety of aldehydes that can participate in carbonyl–amine condensation and aldol condensations, potentially competing for reactive intermediates with the Strecker aldehydes. In addition, studies have shown that carbohydrates can also influence the retention and release of volatile flavour compounds (Naknean and Meenune 2010). Generally, mono- and disaccharides usually increase the vapour pressure, which causes an increase in volatility of flavour compounds relative to water. Polysaccharides, in contrast, incur a reduction in aroma release caused by an increase in viscosity and/or by molecular interactions with flavour compounds (Naknean and Meenune 2010). Proteins have also been shown to bind covalently to thiols and disulphides (Adams et al. 2001) removing, in this way, potent odorants from the system. In addition, the presence of polyphenols can reduce the emission (or amount) of pyrazines (García-Lomillo et al. 2016).

Studying the behaviour of these flavour-matrix interactions will enable us to understand better the dynamics of the formation and release of flavour compounds in food. Moreover, flavour compound-matrix interaction is not only a crucial step in flavour release research, but also is highly relevant for metabolomics/analytical method development. In metabolomics studies, the complexity of food matrices has become a crucial aspect for method development, especially for untargeted analyses.

## Flavour precursors and reaction pathways for aroma-active compound formation

Non-enzymatic reactions occurring in the formation of aroma compounds during food processing include Maillard reactions, caramelization, oxidative and thermal degradation of lipids, as well as degradation of sugars, proteins, ribonucleotides, pigments and vitamins. Once again, the interactions between degradation products can also result in additional chemical reactions. The Maillard reaction, lipid degradation and a combination of both are particularly important for aroma formation in process flavours.

### Maillard reaction

The Maillard reaction, which occurs between amino compounds and reducing sugars, has been recognised for over 60 years as one of the most important routes to flavour and browning formation in cooked food (Shahidi et al. 2014). Louis-Camille Maillard first reported the reactivity of reducing sugars with peptides in 1912 (Maillard 1912). The Maillard reaction is a complex of hundreds of possible reactions. Even with the simplest sugars and amino acids, hundreds of different volatile and non-volatile compounds can be formed. This extremely complex reaction has been the subject of much research by food scientists seeking to discover new mechanisms of formation and to identify new compounds that provide the desired flavour and colour characteristics of heated foods (Nursten 2005; Jaeger et al. 2010; Tamanna and Mahmood 2015). The chemistry has been comprehensively reviewed by Ledl and Schleicher (1990) and more recently by Nursten (2005). Many authors also reviewed the importance of flavour formation through Maillard reactions in different processed foods (Manzocco et al. 2001; Newton et al. 2012).

**Fig. 2:**
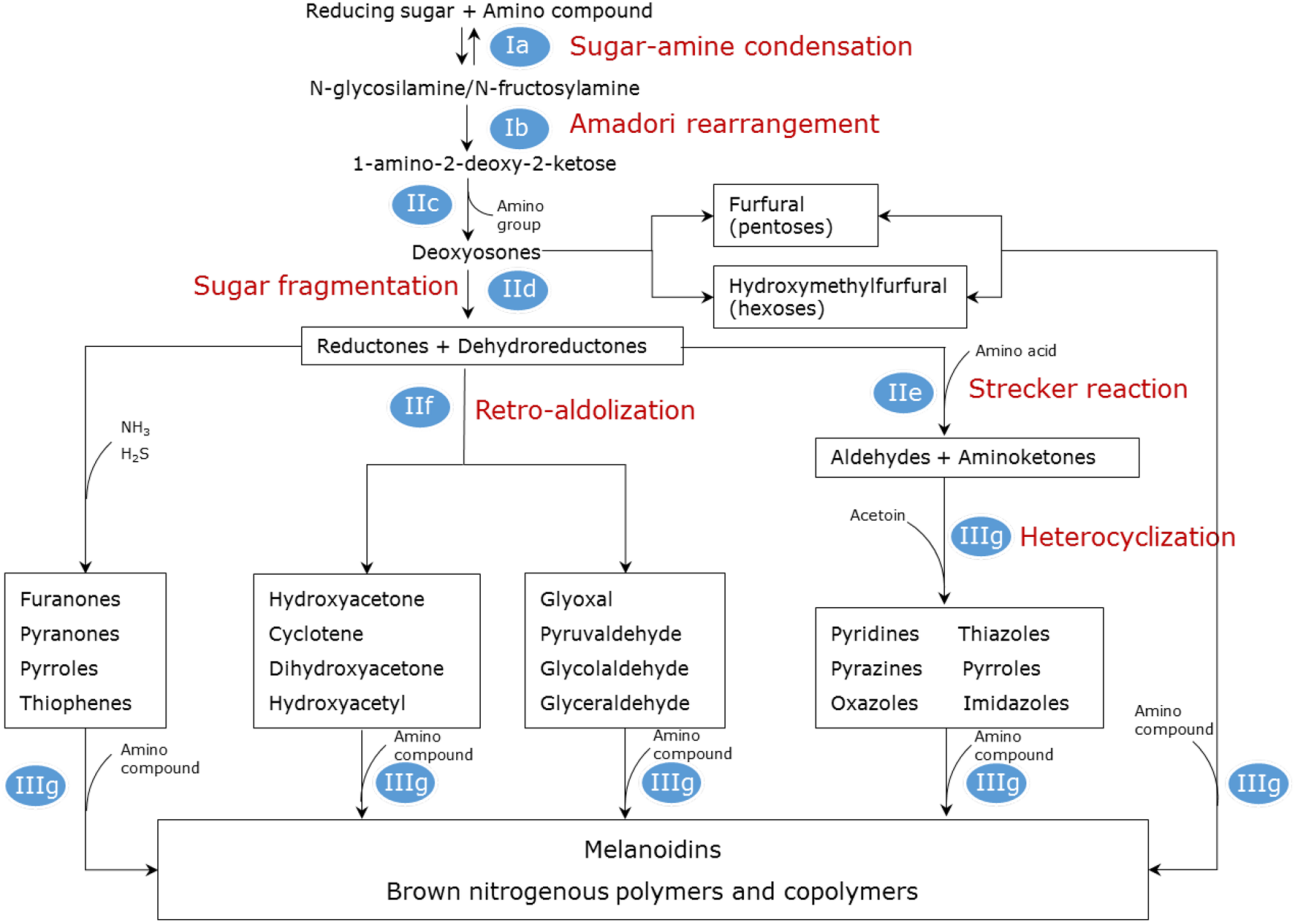
One of the most important sources of typical food metabolites which are of great influence to food flavour and quality arise through usually heat-induced chemical reactions generally grouped under the term ‘Maillard reactions’. Here we present a schematic overview of the Maillard reaction, based on Hodge (1953) and van Boekel (2006), that shows the main-end products contributing to flavour. (I) Early stage (a: sugar-amine condensation; b:amadori rearrangement); (II) Intermediate stage (c: sugar dehydration; d: sugar fragmentation; e: strecker degradation/amino acid degradation); (III) final stage (f: aldol condensation; g: aldehyde-amine condensation and formation of melanoidins)

Hodge (1953) divided the chemistry of the browning reaction into three stages, now generally adopted as the three stages of the Maillard reaction. These are: (i) the early stage (sugar-amine condensation, the Amadori rearrangement); (ii) the intermediate stage (sugar breakdown and dehydration, Strecker degradation) and (iii) the final stage (aldol condensation, aldehyde-amine condensation and formation of heterocyclic nitrogen compounds). Figure 2 shows a schematic overview based on Hodge (1953) and van Boekel (2006). Two reactions occur during the early stage, the condensation of an aldose sugar and an amino compound (*N*-glycosylamine formation), and the rearrangement reaction leading to the Amadori compound (the *N*-substituted 1-amino-2-deoxy-2-ketose) or the Heyns compound if the reducing sugar is a ketose. While the first reaction is reversible, the second is not and is thought to be acid-catalysed. There is no formation of any aroma or colour at this stage. In the intermediate stage, the relatively stable Amadori/Heyns compound can react via two enolisation routes depending on the pH conditions. Sugar fragmentation and release of the amino group occurs, and Strecker aldehydes, among others, are formed (Rizzi 2008); (Fig. 2). These corresponding Strecker aldehydes contain one less carbon atom than the original amino acid (Whitfield 1992) and can be colourless or yellow. They are considered important contributors to the aroma of food products. Many patents have been granted which involve Strecker degradation to produce flavouring materials of foodstuff such as maple syrup, chocolate, coffee, tea, honey, mushroom and bread (Morton et al. 1960). The final stage of the Maillard reaction is comprised of many complex and interconnected reactions of dehydration, fragmentation, cyclization, condensation and polymerisation, in which amino groups again participate. Strecker aldehydes formed from the previous stages can react with each other by an aldol condensation, or they can react with amines at high temperatures to give ‘polymeric’, high molecular mass, coloured products of generally unknown structure, called melanoidins. Heterocyclic ring systems, such as pyridines, pyrazines, pyrroles, and imidazoles, have also been shown to be present in food materials after these reactions (Nursten 2005). However, little is known about their mechanisms of formation.

The control of Maillard products during food processing is essential to prevent the formation of undesired products (like carcinogenic compounds and off-flavours) and to facilitate the production of savoury compounds. For this, we first need to learn more about the chemical reactions and mechanisms during processing conditions. Metabolomics now represents a new approach to help correlate pathway analysis, non-enzymatic conversions and food processing steps (Klevorn and Dean 2018).

### Lipid oxidation

Importantly, lipids influence the aroma and flavour of other components, are precursors of odour and flavour compounds and many even have odours and flavours themselves (Forss 1973). Lipids are generally associated with more negative qualities of food flavour, as they are responsible for rancidity in oils or lipid-containing foods. However, they can also play a positive role as flavour enhancers depending on product characteristics. For example, short chain fatty acids are mainly responsible for rancid flavours in milk whereas the same acids are essential flavour constituents in cheese. Moreover, lipids also play an important role in food texture (i.e. mouth feel) and thus affect consumer attractiveness. During food processing, non-enzymatic (auto)oxidation of lipids may occur, and degradation pathways will lead to the formation of a great number of secondary aroma-related metabolites that can affect flavour.

**Fig. 3:**
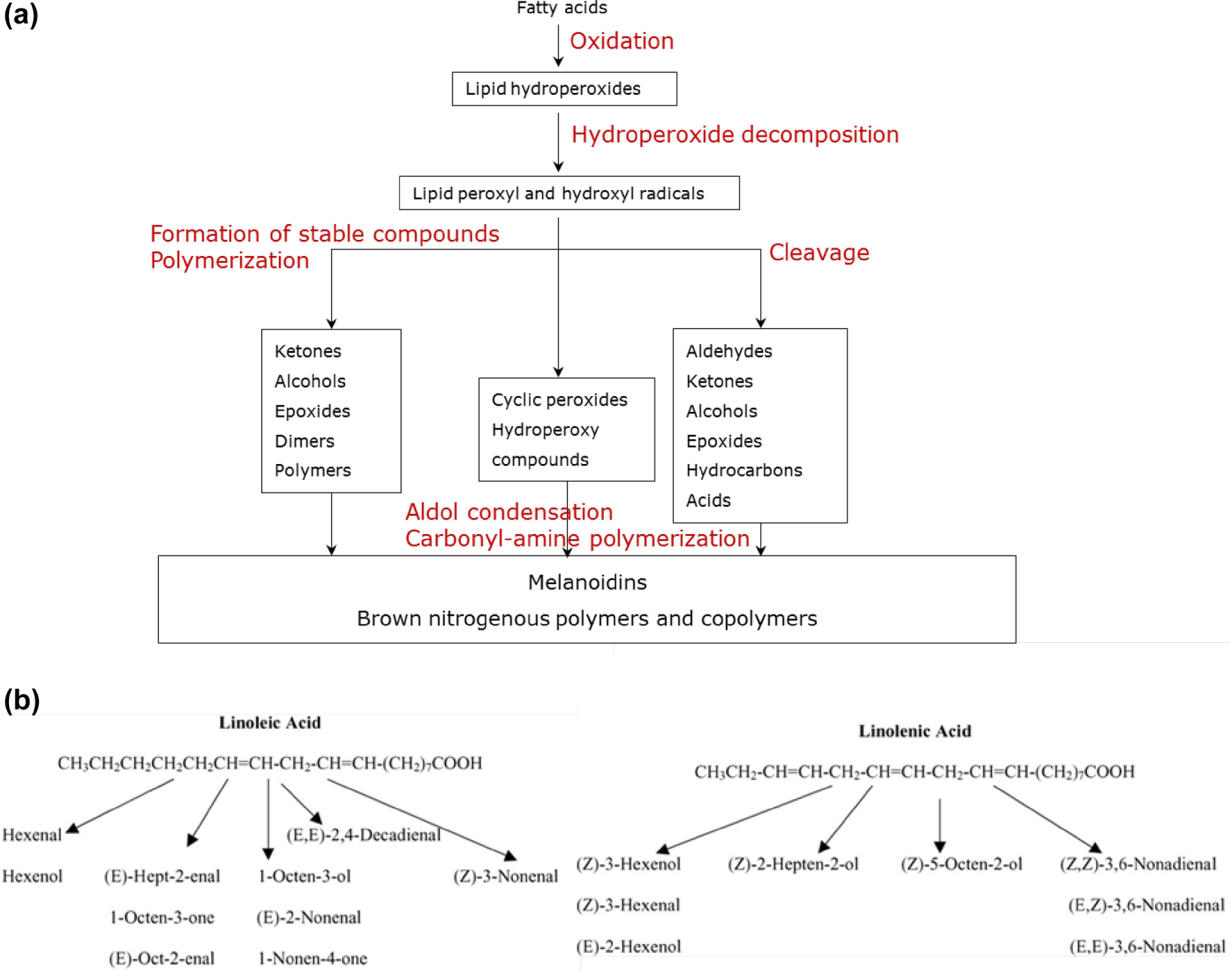
Lipids and their breakdown products are often hugely influential to the overall quality and flavour of food. These compounds can have both a positive or a negative influence on (off-) flavour. Here we present an overview of the lipid degradation pathway: **a** provides an overall picture of the different fatty acid degradation routes and **b** illustrates the complexity using two specific examples of important common fatty acids and their diversity in breakdown products of sensory relevance

Understanding the mechanisms underlying thermal lipid processing needs more attention. Most lipids are hydrophobic, non-polar compounds. Phospholipids and triglycerides are disassembled during heating releasing short-chain fatty acids with reduced saturation. At elevated temperatures, autoxidation of fatty acids occurs and hydroperoxides are produced. This process involves a free radical mechanism which can be divided into three stages: initiation, propagation and termination (Fig. 3a). The initiation reaction is activated by direct thermal dissociation, metal catalysis or exposure to light, forming hydroperoxides (Frankel 1980) which are then decomposed via many routes leading to a broad variety of volatile and non-volatile secondary products. A better understanding of hydroperoxide formation and rearrangement is needed. Advanced metabolomics techniques, such as GC–MS and LC–MS are now being used for the characterization of lipid oxidation products (Xia and Budge 2017). GC–MS analysis provides information regarding the structures of individual oxygenated fatty acids, typically as methyl esters, isolated from oxygenated triacylglycerols (TAGs), while LC–MS techniques allow analysis of intact oxygenated TAGs and yields information on the position of the oxygenated acyl chain on the glycerol backbone (Xia and Budge 2017).

Hydroperoxide decomposition forms an alkoxy radical which mostly transforms into aldehydes, ketones, alcohols and furans. Which products are formed depends on the fatty acids present, the hydroperoxide isomers formed, and the stability of the decomposition products. However, the formation of hydroperoxides is not the only oxidation mechanism involved. According to Schaich (2012), alternative pathways to the hydroperoxide can occur from competing reaction cycles to form peroxides. These peroxides can then either re-enter the traditional propagation stage or undergo alternate reaction pathways thus increasing the complexity of both the kinetics and the product mixture. Most of the research related to food science has been focused on the autoxidation of the most common relevant acids; oleic acid, linoleic acid and linolenic acid. Frankel (1980) and Ho and Chen (1994) list the expected decomposition products from linoleate and linolenate hydroperoxides (Fig. 3b). The rate of autoxidation increases with the degree of unsaturation. Different mixtures of hydroperoxides and their derivatives formed from omega-3 lipids were characterised using SPME-GCMS of oxidized and non-oxidized flaxseed oils (Nieva-Echevarría et al. 2017). An overview of the lipid degradation pathways that lead to flavour formation is shown in Fig. 3a. All of these products are of broad importance in (off)flavour determination—for example, 2,4-Decadienal is known to be one of the most important flavour contributors to deep-fat fried foods.

### Lipid–Maillard interactions

Both lipid degradation and Maillard reactions lead to the formation of a great number of compounds with a similar range of physicochemical properties. It is expected, that these compounds (both intermediate and end products) could further interact to create new volatile compounds and/or block some of the products found in one reaction process by the presence of products from another (Kerth and Miller 2015).

**Fig. 4:**
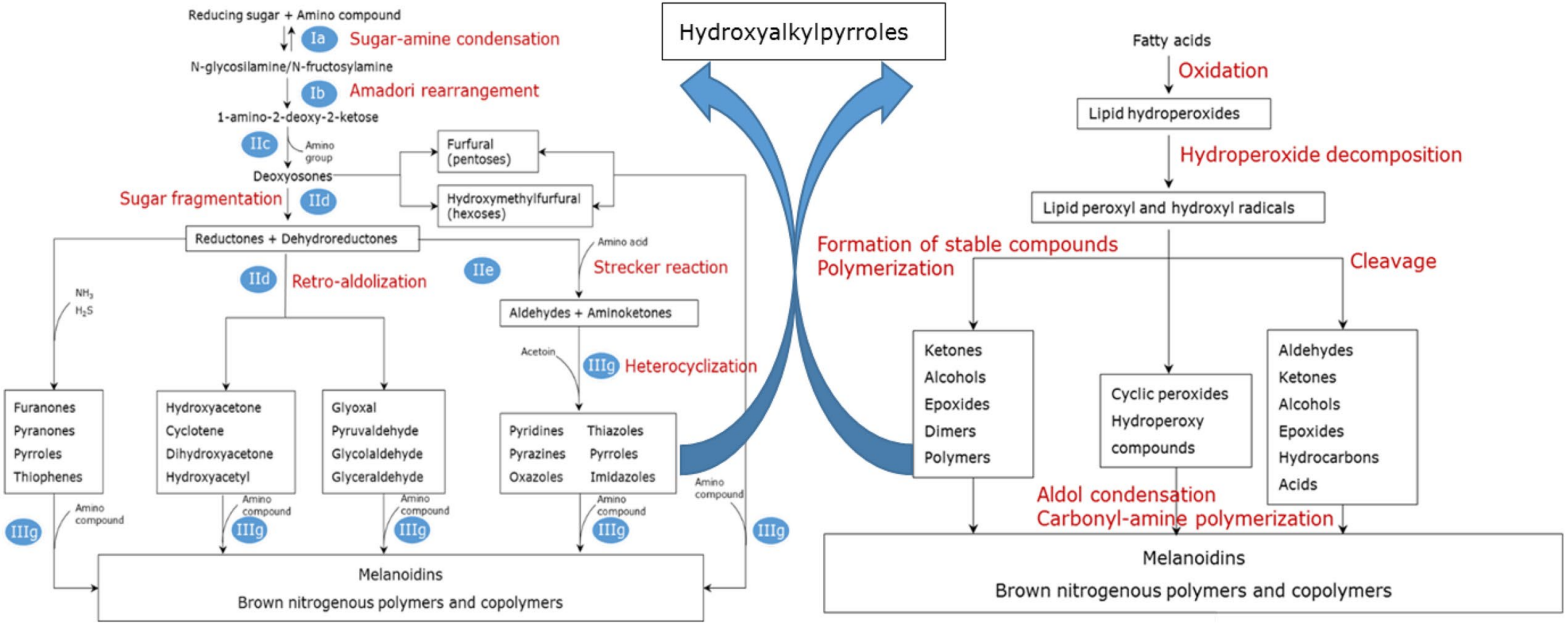
Foods generally have a more complex chemical composition that the fresh materials used for their production. Many new compounds are formed during the main processing steps involving non-enzymic reactions. Here as an example, we show an overview of the common volatile products formed from the interaction of Maillard reaction and lipid autoxidation (modified from Whitfield 1992)

Lipid–Maillard interaction products have been mostly identified in cooked meat, French-fries, peanuts and beverages such as coffee, tea and cocoa. From a review on this topic the largest number of these compounds was found in French-fried potatoes (Whitfield 1992). These were mostly characterized as heterocyclic compounds containing one or more atoms of nitrogen or sulphur, with the presence of long-chain alkyl groups with four or more carbon atoms. Examples of such volatile groups are pyridines, pyrazines, thiophenes, thiazoles and oxazoles with alkyl side chains. Figure 4 shows a few common volatile products formed in this way.

Shahidi et al. (2014) investigated the reactions occurring during the thermal processing of meat. They described how saturated and unsaturated aldehydes, formed via autoxidation of lipids, interact with products from the initial and later stages of the Maillard reaction. Several thiazoles with 4–8 carbon alkyl moieties at the two position have been reported in roast beef along with other alkyl thiazoles. Using GC–MS, longer alkyl chains were identified in the volatiles of heated beef (Elmore et al. 2002). In general, volatile compounds formed from Lipid–Maillard interactions possess weak odour intensities and high odour thresholds. These contribute to the overall aroma at a lower level compared to compounds generated in the primary reactions. The strongest effect on the flavour profile when Lipid–Maillard interactions are present is due to the variation on the Maillard reaction products as affected by lipids (indirect impact on the aroma profile). In particular, Shahidi et al. (2014) reported that phospholipids and their degradation products inhibit important reactions involved in the formation of heterocyclic aroma compounds in the Maillard reaction. Therefore, in the case of cooked food, this interaction may help to maintain the concentrations of sulphur compounds at an optimum level. On the other hand, Elmore et al. (2002), using meat-like model systems, were able to detect the formation of alcohols and alkylfurans instead of the saturated and unsaturated aldehydes when polyunsaturated fatty acids were present. Possible pathways for the formation of those compounds have been proposed. However, many gaps in our knowledge are still present.

### Others

**Fig. 5:**
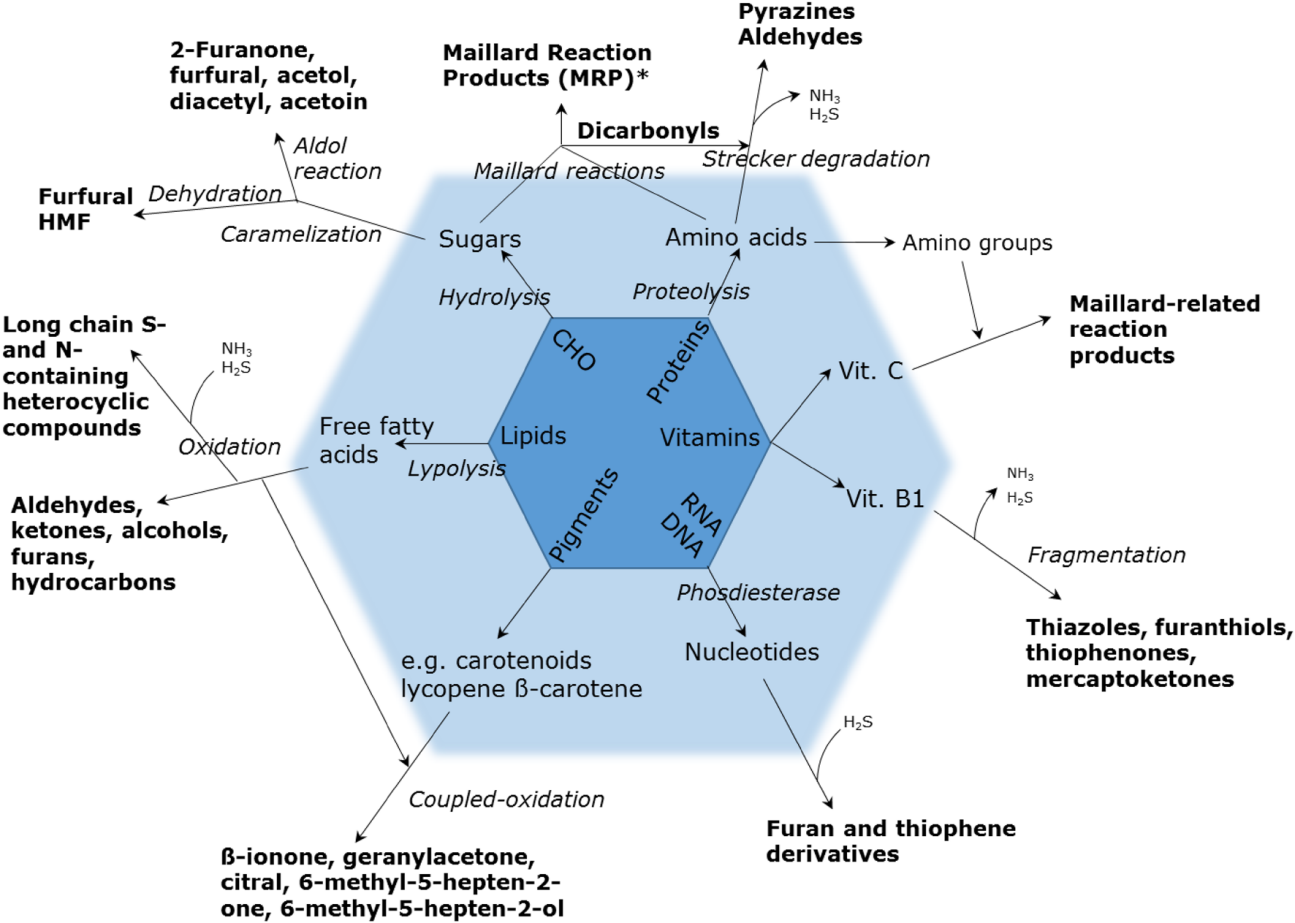
An overview of the chemical reactions relevant to flavour formation, modified from Sucan and Weerasinghe (2005). The typical complexity of the volatile components in food products is the result of both the modification of the original chemical constituents of the raw materials during processing as well as the subsequent interactions within and between the different chemical groups catalysed by enzymatic and thermo/chemical reactions

Apart from the great proportion of aroma compounds derived from sugars, amino acids and lipids, there are also other essential compounds found in processed food which can play an important role in flavour formation. Thiamine (Vitamin B1), ascorbic acid (Vitamin C) and carotenoids are examples of such compounds. During thermal processing, these compounds can undergo degradation processes and contribute to the formation of odour-active compounds. Thermal breakdown of these products produce reactive intermediates that are common to the Maillard reaction. Therefore, thiamine degradation leads to the formation of S-containing heterocycles, thiols, sulphides and disulphides that contribute to a meaty flavour in cooked products (Khan et al. 2015). A schematic compilation of the degradation pathways that lead to aroma formation, from the primary precursors, is illustrated in Fig. 5. Recently, Yu and Zhang (2010) described the volatiles generated when ascorbic acid and cysteine were heated in aqueous buffer at different pHs. Many of these were compounds that can contribute to meat flavour such as thiophenes, thiazoles, pyrazines and cyclic sulphur compounds. However, using dynamic headspace (DHS) extraction rather than SPME, Parker (2015) showed that 2-methyl-3-furanthiol and many related disulphides were formed in buffered model systems containing ascorbic acid and cysteine.

Another important reaction in processed food is caramelisation. This thermal degradation of sugars occurs in the absence of amino acids, and the products that are formed are similar to those of the Maillard reaction (Fig. 5). However, when an amino group is present it acts as a catalyst resulting in a faster reaction and higher amounts of very reactive intermediate products (Van Boekel 2006).

## Volatiles contributing to flavour

### Aldehydes, alcohols and ketones

Aldehydes, alcohols and ketones are groups of chemical compounds that play a key role in the overall flavour of processed foods and e.g. are responsible for the characteristic aroma of fermented foods (Visessanguan et al. 2006; Gambacorta et al. 2009). Moreover, many aldehydes are also naturally present in fresh foods, such as fruits and vegetables, as well as in essential oils. Concentrations increase after thermal processing due to chemical reactions at high temperatures. They can also be formed by oxidation of unsaturated fatty acids, by normal fatty acid metabolism or by the conversion of amino acids (Figs. 2, 3, 4). These molecules cover a broad range of different chemical and physical properties and hence, different analytical approaches are needed. Smaller aldehydes, such as formaldehyde and acetaldehyde, are more soluble in water. GC–MS and LC–MS approaches have been developed to identify short-chain aldehydes. One of the earliest detection techniques for fatty aldehydes was their analysis by GC as dimethylacetal derivatives (Berdyshev 2011). However, headspace extractions are now commonly used. On the other hand, LC–MS approaches have the advantage of the higher stability of hydroxy-aldehydes as compared with the higher temperatures used in GC–MS.

Soy sauce aroma is commonly used in food processing as a flavouring. Its main flavour attributes are based on Maillard and fermentation processes. Aldehydes, alcohols and ketones were the main volatile compounds found in soy sauce products (Fan et al. 2011; Zheng et al. 2013). Several methods have been used for the extraction of aldehydes, alcohols and ketones in soy sauce, such as liquid–liquid extraction (LLE) (Xu et al. 2007) and solid-phase microextraction (SPME) (Gao et al. 2010; Chen et al. 2015). More recently, stir bar sorptive extraction (SBSE) has been tested in Chinese soy sauce aroma type liquor for the analysis of volatile compounds (Fan et al. 2011). SBSE is an environmentally-friendly alternative characterized by its ease of use, high selectivity, high sensitivity, and reproducibility. Fan et al. (2011) also optimized the SBSE method for the analysis of the overall aroma volatile profile of soy sauce liquors, including esters, alcohols, aldehydes and ketones, aromatic compounds, furans, nitrogen and sulphide-containing compounds, acids, phenols and terpenes. Table 1 shows a summary of some characteristics from the different chemical groups explained throughout Sect. 4.

### Organic acids and derived esters

Volatile esters are commonly found in many food products. Aliphatic esters are very important constituents of many process flavours as they give very intense fruity notes. Esters are often characterized as contributing ‘banana flavour’ (isoamyl acetate) or ‘apple flavour’ (ethyl hexanoate, ethyl octanoate). Ethyl acetate is often characterized by an ‘adhesive’ flavour note. Ethyl butyrate, valerate, hexanoate and octanoate are all considered to have strong flavours.

Esters are formed from lipid metabolism (Fig. 3). Lipids are converted to a large number of alcohols and acids that may then undergo esterification. Esters are also present in process flavours, but at low concentrations and thus have little effect on the whole aroma. Lin et al. (2014) identified two esters (acetic acid ethenyl ester and (S)-2-hydroxy propanoic acid ethyl ester) in yeast extract pastes using simultaneous distillation extraction, and detected by combined GC–O/MS.

### Nitrogen- and oxygen-containing heterocyclic compounds

Heterocyclic compounds are found at relatively low levels in food. However, they do add a significant degree of complexity to food and, thus, they boost the overall flavour of a product and increase its desirability to consumers. Heterocyclic compounds are strongly related to roast meat flavour formation during heating and are important compounds in food processing and flavouring. They have been observed in reaction mixtures of amino acids and sugars but some can also be formed from lipids (Whitfield 1992). Furans, pyrroles, pyridines, pyrazines, oxazoles and oxazolines are typical volatile N- and O-containing heterocyclic compounds which are mainly derived from the reaction between cysteine and xylose (Cao et al. 2017). Apart from sulphur-containing compounds, heterocycles such as methylfuran, pyrazine, and furfural are recognized as potent meaty flavour compounds. Oxazoles, oxazolines, pyrroles and pyridines have also been identified in cooked meats (Devine and Dikeman 2014). However, their contribution to the overall aroma is not as significant as that of sulphur-containing compounds which possess closely related chemical structures, such as thiazoles and thiazolines (See next section).

#### Furan-derivatives

Furan-derivatives have a planar enol-carbonyl structure in a cyclic dicarbonyl compound. They originate from the early stages of the Maillard reaction and deliver caramel-like aromas (Shahidi et al. 2014). These compounds are also important intermediates in the formation of other N- and S-containing flavour volatiles as they can easily exchange oxygen with nitrogen and sulphur. Examples of furan-derivatives are illustrated in Fig. 4. There are multiple pathways underlying furan and furan-derivative formation. According to Seok et al. (2015), furan is formed from (i) thermal degradation or rearrangement of carbohydrates alone, or in the presence of amino acids, (ii) thermal degradation of certain amino acids, (iii) oxidation of ascorbic acid under high temperatures, and/or (iv) oxidation of polyunsaturated fatty acids and carotenoids (see Figs. 2, 3, 4).

Furan-derivatives are important flavouring compounds and their characterization and identification by analytical methods is of great relevance for designing and producing high-quality food products. An important food ingredient used as seasoning and widely consumed around the world is soy sauce. Over 300 volatile compounds have already been identified in soy sauce (Kaneko et al. 2012) with furan-derivatives being the most abundant and sensory-relevant compounds. Kaneko et al. (2013) investigated the volatiles of soy sauce and how these change on heating. Aroma extraction dilution analysis (AEDA) was used. This is the most frequently applied method for the screening of flavour-impact compounds when using GC–O. During AEDA, the original flavour extract is sequentially diluted and the diluted extracts are then evaluated by GC–O to provide flavour dilution (FD) factors. These dilutions are usually combined with extraction techniques using solvents, such as liquid–liquid extraction (LLE), simultaneous distillation/extraction (SDE), or solvent-assisted flavour evaporation (SAFE) (Curioni and Bosset 2002; Poehlmann and Schieberle 2013; Munafo et al. 2014) and recently with SPME (Feng et al. 2015). Several parameters, including fibre type, extraction (exposure) time/temperature, and the saturation of the aqueous phase in the headspace vial, affect optimal SPME conditions. Some studies focused on the selection of optimal fibers for the analysis of furan (Seok et al. 2015) and report the best option is a carboxen/polydimethyl-siloxane (CAR/PDMS) fiber, as this showed marked advantages such as selectivity.

#### Pyrazines

Pyrazines represent an important component of process flavours and constitute a major class of volatiles formed via the Maillard reaction, conferring a ‘roasted flavour’ character. Currently, pyrazines are included in the list of flavouring agents authorized by the European Union which may be incorporated into food products to imitate meat flavours (García-Lomillo et al. 2016). However, the majority of the pyrazines in food are naturally occurring and often have a very low odour threshold. The common structure of pyrazines is a six-membered aromatic ring with two nitrogens in *para* position. They differ in the substituents at one or more of the four ring carbon atoms. Alkyl and alkoxy pyrazines are the most predominant forms used in process flavours. A few examples are illustrated in Fig. 5. Alkyl pyrazines can help enhance the flavour of cooked foods by adding a savoury taste to the overall product. They are characterized by a roasted, nutty, cocoa, sweet flavour. Alkoxy pyrazines are also commonly used to add a characteristic savoury, and sometimes, spicy aroma to a number of different products. Müller and Rappert (2010) describe the occurrence, formation and biodegradation of pyrazines as used in flavourings. Pyrazines are routinely analysed by headspace GC–MS because of their volatility. However, organic extractions are also used (Lin et al. 2014). They compared the analysis of the volatile content of two different yeast extract pastes by using dynamic headspace and simultaneous distillation extraction (SDE). SDE extracted and detected more pyrazines than DHS and is hence to be preferred. Adams et al. (2008) used SPME-GC–MS for the analysis of different pyrazines formed from several model reactions and also discovered the formation of a novel pyrrole in the model reaction of alanine with 2-oxopropanal.

### Sulphur-containing compounds

Sulphur-containing compounds are among the most important aroma volatiles in many processed foods (Landaud et al. 2008; McGorrin 2011). Both aliphatic and heterocyclic sulphur volatiles are present in food at low concentrations, but their low odour thresholds make them potent aroma compounds conferring sulphurous, onion-like and meaty aromas to foods. Sulphur compounds are volatile and many GC–MS techniques have been used for the detection of these compounds, mostly following solvent extraction. However headspace techniques have also been developed for the qualitative and quantitative analysis of these compounds as shown below.

#### Alkyl sulphides and polysulphides

*Allium* vegetables and their organosulphur compounds are possible cancer-preventative agents in humans as they appear to inhibit the formation of a range of different cancer types (Omar and Al-Wabel 2010). Alkyl sulphides and polysulphides are important compounds for the aroma of many flavouring agents. Polysulphides are formed from the thermal degradation of thiamine and components of the methionine and cysteine pathways via the Maillard reaction (Fig. 2). They contribute to a cooked meat aroma but, at high concentrations they can give food an off-flavour. Many studies have identified these compounds in garlic and onion samples (Jones et al. 2004; Tocmo et al. 2017). They are created by the degradation of relatively stable, odourless, S-alk(en)yl cysteine sulphoxide flavour precursors and these polysulphides are known to be unstable, depending on the extraction technique. Lee et al. (2003) compared several sampling techniques for the determination of Korean cut garlic flavour components by GC–MS including steam distillation (SD), simultaneous distillation and solvent extraction (SDE), solid-phase trapping solvent extraction (SPTE), and headspace solid-phase microextraction (HS-SPME). Thermal degradation of components such as allyl methyl sulphide, dimethyl disulphide, and thiirane were observed for SDE and SD but not for SPTE or HS-SPME. HS-SPME had several advantages compared with SD, SDE, and SPTE such as rapidity, no apparent thermal degradation, is less labour intensive, and small sample size (Lee et al. 2003). Five different fibre coatings were also evaluated for HS-SPME of garlic flavour components. DVB/CAR/PDMS was the most efficient of the five types investigated. However, some researchers report limitations on the use of SPME if quantification is desired (Murray 2001).

Many vegetables, when cooked, are used as natural flavourings in a wide range of dishes. Garlic (and other species from the genus *Allium*) and ginger are routinely used in stir-fried Chinese dishes. The most important precursor of garlic flavour is allicin (allyl 2-propenethiosulfinate). Allicin is very unstable and readily degrades into other secondary sulphur compounds and a variety of sulphides, when garlic is crushed and then heated. The sulphur compounds thus formed contribute to the pungent flavour of cooked garlic. It was found that in a GC column, allicin will decompose into 3-vinyl-[4H]-1,2-dithiin, 2-vinyl-[4H]-1,3-dithiin, and a few trace compounds (Ho and Chen 1994). The major compounds of heated garlic oil have been identified and are likely to be sensory-relevant. On the other hand, Colina-Coca et al. (2013) reported the analysis of many alkyl sulphides, disulphides and trisulphides by dynamic headspace (DHS) and GC–MS in processed onion. They found differences in the aroma and volatile content of the samples according to the way the onion was processed (raw, high pressure processing). Tocmo et al. (2017) also reported the analysis and identification of alkyl polysulphides by organic extractions.

#### Thiophenes

Thiophenes are important Maillard products which confer a desirable ‘meaty’ aroma to food. Thiophenes with a thiol group at the three-position possess a strong meaty-like aroma and have low odour threshold values. There are a number of possible routes to the formation of thiophenes, involving the reaction of a sulphur-containing amino acid (e.g., cysteine, cystine, methionine) or thiamine, with intermediary sugar degradation products coming from the Maillard reaction (See Figs. 2, 4). Lin et al. (2014) reported the detection of 5-methyl-2-thiophenecarboxaldehyde only detected by dynamic headspace/GCMS as compared to simultaneous distillation analysis in yeast extracts used as flavourings. Similarly, Mahadevan and Farmer (2006) reported the detection of some thiophenes, aliphatic sulphur compounds, and cyclic polysulphur compounds collected by dynamic headspace concentration of yeast extract pastes. On the other hand, static headspace (such as the traditional SPME), combined with two dimensional gas chromatography allowed the identification of 23 thiophenes in roast beef (Cordero et al. 2015) that could be used as aroma compounds in process flavours.

#### Thiazoles and thiazolines

Thiazoles and thiazolines are important compounds in roasted or fried meat and have an important role in flavour chemistry. Similar to pyrazines, their content increases with higher cooking temperatures and they are potent flavour ingredients with low aroma thresholds. Most thiazoles contributing to meat flavour are alkyl substituted, and their specific aroma depends on the nature and the number of alkyl moieties attached. One possible route for the formation of thiazoles and thiazolines is via the Maillard reaction involving the action of ammonia and hydrogen sulphide in the presence of α-carbonyls, dicarbonyls, or hydroxyketones (Fig. 2) as derived from the Strecker reaction. However, lipid-derived aldehydes can also participate in this reaction during cooking (Fig. 4) and long-chain trialkyl thiazoles have been extracted from meat by dynamic headspace methods and identified by GC–MS (Elmore et al. 1997; Shahidi et al. 2014). In process flavours made from yeast extracts, many thiazoles were detected using the same technique (DHS/GC–MS) (Ames and Elmore 1992). Yeast is a rich source of thiamine and thus, it can act as precursor for thiazoles and thiazolines.

## Sensory analyses and prediction models

### Definition of flavour, taste, odour

The definition of flavour is the overall sensation resulting from the impact of food on the chemical sense receptors in the nose and mouth (Dresow and Böhm 2009). Soluble, non-volatile substances released from food stimulate taste receptor cells, while the volatiles released reach the nose epithelium to give the odour sensation through the so called retro-nasal pathway (Guichard and Salles 2016). The combination of taste and odour is termed flavour. However, the most important contributor to flavour is odour. This becomes evident when a person catches a cold and cannot sense flavour by the nose. Only 200–400 volatiles among 10,000 identified volatile compounds in food are proposed to determine the characteristic odour of a food product (Dunkel et al. 2014). Analyses demonstrated that some very important aromas are not the result of the presence of a unique characterizing compound, but rather result from a reproducible blend of a particular number of components in a specific balance (Dresow and Böhm 2009).

### Sensory analysis and prediction models in food processing

Sugars contribute to sweet taste, and acids to sour taste exclusively, the amino acids and simple peptides can elicit all five primary taste sensations (Sucan and Weerasinghe 2005). Sensory analysis is a scientific discipline that evaluates consumer products by using human senses (sight, smell, taste, touch and hearing) in a complete experimental design (Lawless and Heymann 2010). Panels of trained human assessors are required and, by applying statistical analysis to the results it is possible to make inferences and insights about the products under test.

When designing a food product, it is important to relate the chemical composition with sensory attributes. By knowing the compounds that cause a specific flavour characteristic, we are able to identify the chemical pathway and hence, the flavour precursors that may be ‘activated’ for product formulation. Thus, a good correlation between the chemical profile and the sensory profile must be determined and hence knowledge of the chemical pathways of flavour formation is needed. While there are some attractive ‘proof-of-concept’ examples reported for certain (fresh) food products, such chemometric/multivariate modelling approaches have yet to be applied for process flavours. Prediction modelling of sensory profiles and dynamic modelling of (off-)flavour formation is becoming an important research area in food science. Attempts to relate sensory analysis data to specific chemicals such as volatile compounds have been frequently reported (Lubes and Goodarzi 2017; Seisonen et al. 2016). A non-targeted chemometric approach was use to successfully identify ethyl acetate as a specific off-flavour compound in poor-quality coffee samples (Lindinger et al. 2009). Tikunov et al. (2013) also use a combination of genomic and transcriptomic analysis together with untargeted GCMS-based metabolomics to identify the gene behind the smoky flavour of tomato. Subsequent blind taste panel and spiking experiments confirmed a causal link between a small number of phenylpropanoid volatiles and the smoky sensory trait. Chemometric analysis has been exploited frequently in common processed and fresh foodstuffs such as olive oil (Procida et al. 2016) and apple (Aprea et al. 2012; Corollaro et al. 2014). However, these associations can often be weak or difficult to interpret. This may be due to the chemometric method used and/or the natural complexity of food and its flavour. Moreover, current prediction models are also limited to a small number of targeted individual compounds, involve single analytical techniques, or are only focused on specific sensory attributes. As a consequence, they are not robust enough and/or do not allow the generation of novel hypotheses for changing product formulation and manufacturing processes. Expanding the prediction models to a large number of compounds and multiple sensory attributes entails both analytical and statistical challenges.

Chambers and Koppel (2013) reviewed some of the reasons sensory analysis and instrumental measurements often result in poor associations and have identified issues that need to be addressed in future research into understanding the relationships between flavour/aroma phenomena and chemical composition. One difficulty in modelling volatile odour characteristics is that the perception of flavour can vary when volatiles are present in different food matrices, which in itself by definition also remains subjective. For example, hexanal has often been positively associated regarding green/grassy odour. However, negative associations have also been found, with rancidity and oxidized aromatics.

In the case of process flavours, very little has been done in relation to the volatile chemical composition with the sensory data. Thus, the use of supervised and unsupervised statistical techniques should be put into practice for these more complex flavourings, as was followed by Zielinski et al. (2014).

## Conclusions

### The importance of (volatile) secondary metabolites in food

Volatiles are only a small proportion of the total number of metabolites in food yet their contribution to food flavour makes them crucial compounds for understanding food science. For example, a rice grain contains 92% starch and 7% protein but the huge difference in market value and consumer preference relates to the remaining 1% including the volatile components (Calingacion et al. 2015). Furthermore cooked food contains many more volatiles than the raw material (Belitz et al. 2008) and it is this complexity which calls for a metabolomics approach to advance our knowledge. Food metabolomics is helping to reveal the mechanisms underlying volatile flavour formation and subsequently the precursor chemical pathways involved. Faster, more sensitive and more comprehensive analysis of food is now possible. Nevertheless, challenges remain—for example, correlating human sensory panel and metabolomic data. In order to innovate, it is important to understand how to make ingredients and food healthier, palatable and more profitable across the entire processing chain. Maintaining positive flavour attributes along the supply chain is one approach to produce better and tastier food.

### The major challenges of food metabolomics

The overall metabolite analysis of the complex matrices typical of processed foods is one of the biggest challenges in food metabolomics. Advances in chromatographic separation and data processing are greatly advancing our understanding of process flavours and how these relate to food processing strategies. However, there are still many limitations regarding the separation methods for these complex matrices (Scalbert et al. 2009). Yang et al. (2015) reported a data pre-processing strategy to reduce the masking effect of complex sample matrices. For untargeted analysis, the aim is to detect as many components of matrix as possible. Therefore, metabolite analysis without using solvents results in a much higher (unspecific) coverage of the different chemical groups, as well as a fast, sensitive, solventless and economical alternative. Traditional techniques such as SPME are still being improved thanks to advances in high-throughput instrumentation and data pre-processing (Kataoka et al. 2000). On the other hand, emerging technologies such as SBSE techniques are good alternatives as they require little sample preparation (David and Sandra 2007; Camino-Sánchez et al. 2014; Nogueira 2015). There is no singular method that allows for accurate, sensitive and complete reporting of chemical species but with these new analytical developments increased comprehensiveness is emerging.

Low abundant compounds are difficult to detect. However, in food, these can greatly influence the sensory properties when they also have low aroma thresholds. For instance, volatile sulphur compounds are characterised by their low odour threshold and strong reactivity, making these compounds of great importance to the quality and uniqueness of foodstuffs like cheese, beer, wine and meat (McGorrin 2011). Concentration levels are sometimes below the capacity of the analytical technique indicating that analytical sensitivity can be lower than human detectability. Odour thresholds play an important role in sensory analysis. Some compounds result in intensive aromas even at trace concentrations, while other compounds can change aroma profiles at differing concentrations. Furthermore, food matrices can also influence the perception of individual compounds as compared to the overall flavour of the mixture.

Untargeted food metabolomics is also currently limited by the identification of unknowns. The lack of available libraries with food metabolites makes rapid identification a challenge. In untargeted metabolomics studies, major improvements are still required for spectral deconvolution and automatic metabolite identification. Although major efforts are being made to improve spectral databases, the development of accurate automatic identification algorithms is still subject to the availability of an exhaustive set of reference metabolites (Alonso et al. 2015). The identification of unknowns even present in trace quantities in natural foods is essential for novel processing strategies to deliver superior food flavour and quality. In addition, the identification of new ‘natural’ flavour compounds in cooked and roasted foods would doubtless be of great interest to the flavour industry.
